# Current biomarker-associated procedures of cancer modeling-a reference in the context of IDH1 mutant glioma

**DOI:** 10.1038/s41419-020-03196-0

**Published:** 2020-11-21

**Authors:** Narges Zare Mehrjardi, Daniel Hänggi, Ulf Dietrich Kahlert

**Affiliations:** Clinic for Neurosurgery, Medical Faculty Heinrich-Heine University, Moorenstrasse 5, 40225 Duesseldorf, Germany

**Keywords:** Mechanisms of disease, DNA metabolism, Diagnostic markers

## Abstract

Isocitrate dehydrogenases (IDH1/2) are central molecular markers for glioblastoma. Providing in vitro or in vivo models with mutated IDH1/2 can help prepare facilities to understand the biology of these mutated genes as glioma markers, as well as help, improve therapeutic strategies. In this review, we first summarize the biology principles of IDH and its mutations and outline the core primary findings in the clinical context of neuro-oncology. Given the extensive research interest and exciting developments in current stem cell biology and genome editing, the central part of the manuscript is dedicated to introducing various routes of disease modeling strategies of IDH mutation (IDH^Mut^) glioma and comparing the scientific-technological findings from the field using different engineering methods. Lastly, by giving our perspective on the benefits and limitations of patient-derived and donor-derived disease modeling respectively, we aim to propose leading research questions to be answered in the context of IDH1 and glioma.

## Facts

- Mutations in IDH genes represent the first central genomic markers to guide clinical diagnosis in neuro-oncology.
- The involvement of IDH mutations in various processes of glioma cell biology opens an opportunity for new treatment and diagnostic strategies, with several clinical trials underway.
- Classical in vitro disease modeling in the context of IDH mutant brain tumors using patient-derived systems is challenging given the general disadvantages in terms of cell growth and survival.
- Novel strategy of in vitro cancer modeling using the transformation of healthy donor-derived stem cells is emerging to combat this hurdle.

## Open questions

- Can single cell-derived, synthetic cancer in vitro modeling based on the transformation of healthy donor-derived cells with disease resembling genetic elements overcome hurdles experienced when relying on cancer cell lines only (i.e. limitations in reproducibly of research results, a limited accurate recapitulation of pathophysiology)?
- Since IDH1 mutations occur in other cancers than brain tumor, can the progress of understanding the glioma IDH1 mutation cell biology translate into tumor-agnostic approaches?
- Can the interrogation of technological advanced cell model systems in in vitro screening approaches justify the prolonged establishment of such explorative experimental design in the early stage of current drug development processes?

## Introduction

Mutations in the DNA encoding for isocitrate dehydrogenases (IDH) mutations are recognized as one of the main molecular markers in 70–80% of stage II or III astrocytomas, oligodendrogliomas, and secondary GBMs, as opposed to primary tumors^1^. In the WHO classification, expressions of mutated IDH1/2, TP53, chromatin remodeling, and a loss of alpha-thalassemia/mental retardation, X-linked (ATRX) has been seen in astrocytoma. In contrast, oligodendrogliomas carry mutated IDH1/2 cells associated with 1p/19q co-deletion and TERT promoter mutations^2,3^. The biology of IDHs in cancer is not fully understood, but several therapeutic opportunities arise as a result of this molecular alteration. To better understand and develop precision medicine approaches to IDH mutation (IDH^Mut^) relevant modeling tools are needed. Despite the technical advances in modern biomedicine, the field is suffering from a lack of pathophysiological relevant modeling systems to study the disease in a controlled experimental setting. In this review, we focus on current disease modeling efforts for one of the most studied genes linked to deadly cancer. To our knowledge, this is the hitherto most comprehensive reference describing technical details of successful cell engineering protocols that assess their practicality and applicably from an academic lab perspective. By including a listing of the main biological findings made with different lab tools and comparing them to the patient scenario, we also propose a ranking of their pathophysiological relevance. Given the comprehensive nature of our review that examines the details of current stem cell technologies, molecular editing, and in vitro pharmaco-metabologenomics, we anticipate that the significance of this review will extend well beyond the field of neuro-oncology.

## Principles of IDH function and pathological findings in a clinical context of IDH1 mutation (IDH1^Mut^) in neuro-oncology

IDH is a small protein that expresses primarily in the liver, heart muscle, and skeletal muscle^4^. There are five genes that encode IDHs: IDH1, IDH2, IDH3A, IDH3B, and IDH3G. IDH1 and IDH2 are active as homodimers, while IDH3 is active as a heterotetramer containing 2α, 1β, and 1γ subunits, all of which function as the electron acceptor^5^. The role of mutated IDH1/2 has been reported in low-grade gliomas (LGG)^6^. According to the authors’ knowledge, no study shows the IDH3 mutation related to any kind of cancer. For the first time, in 2006, Sjoblom et al.^7^ reported the IDH1 mutation (IDH1R132C) in breast and colorectal cancers. Two years later, Parsons et al.^8^ announced an IDH1-R132H mutation in gliomas. Mutated IDH1, as a common mutation, was then reported in glioma, acute myeloid leukemia (AML), cholangiocarcinoma, melanoma, and cartilaginous tumors^8^.

IDH mutations in gliomas are normally heterozygous missense mutations^9^. In addition to the R132H mutation, other IDH1 mutations in glioma are R132L, R132C, R132G, R132S^10^, R132V^5^, and IDH2-R172K or R140Q^5,11^ which all of these mutations show identical output. However, a mutation in IDH1 or IDH2 is considered an early event in gliomagenesis^12^; they are not classic oncogenes. It seems that these genes can facilitate pro-oncogene mutations, such as TP53 in IDH1-R132H astrocytoma^13^, or downregulation of the expression of the genes related to immune response, which was observed in IDH1-R132H gliomas^14^. The protein of IDH1 localizes in the cytoplasm and peroxisome, while IDH2 localizes in the mitochondria. These two genes are (NADP1)-dependent and promote the oxidative decarboxylation of isocitrate to alpha-ketoglutarate (α-KG) that protects the cells from reactive oxygen species (ROS), which can cause DNA damage^15^. In addition, α-KG has been shown to serve as a cofactor for several important cellular reactions, including histone modifications, hypoxia sensing, and fatty acid metabolism^16^. Any mutation in IDH1 or IDH2 forces the cells to convert a-KG into the D isomer of 2-hydroxyglutarate (D2HG)^17–20^. Due to the effect of the D2HG in cancer, this metabolite has been called oncometabolite which can cause several epigenetic abnormalities, such as DNA demethylases, histone modification, non-coding RNA (ncRNA), microRNA (miRNA), and chromatin remodeling^21^. Among IDH1 mutations, R132H is the most common mutation (90%) in glioma^22^. In this mutation, adenine is replaced with guanine at nucleotide 395 (c.395G>A), which converts to histidine instead of arginine in protein sequencing (p.Arg132His). Pusch et al.^23^ clarified that this event is due to the fact that human glioma cells with R132G, R132C, and R132S produce higher concentrations of D2HG as compared to those with R132H. A high concentration of D2HG is toxic for the glioma cells and induces biological alternations such as inhibiting the proliferation and migration of these cells. Therefore, cells prefer R132H mutation with a moderate amount of D2HG^23^. The concentration of D2HG in glioma cells carrying IDH1-R132H is usually between 10 and 30 mM^24^. In addition to glioma cells, D2HG can be found in the non-neoplastic cells around the tumors in the patients carrying IDH1 mutation. Linninger et al.^24^ detected around 100 mM of D2HG in the circulating cerebrospinal fluid (CSF) of LGG patient. This amount of D2HG can cause oxidative stress, inhibition of expression of pro-apoptotic proteins, reduction of pro-inflammatory signaling, and changing cellular metabolism in these patients^24^. Jin et al.^18^ have shown that the level of the D2HG cells carrying IDH1 mutation depends on the wild-type (wt) allele, however; this level in IDH2 mutated cells is related to the site of mutation. Ward et al.^25^ revealed that glioma cells carrying IDH2-R140K produce less D2HG in comparison to IDH2-R172K mutation. Although the role of heterozygous IDH1 mutation (IDH1wt-R132H) is known in glioma biology, the association of the level of D2HG and localization of it in the subcellular compartment on the abnormality of the brain cells in glioma patients is little understood^26^.

Recently, the cancer genome atlas (TCGA) analysis by Unruh et al. showed that IDH1wt/R132H has a special effect on the DNA-methylome and transcriptome of gliomas. A study of DNA-methylation of the patient samples with glioma demonstrated that from 365,092 analyzed CpG sites, 70,591 (19%) were hypermethylated in gliomas carrying IDH1wt/R132H, compared to wild-type gliomas. The ratio of hypermethylation changes during differentiation leads to suppression of tissue development^12^ and differentiation^27^ of glioma cells. In addition, this mutation can hypermethylate and modify important glioma-related genes, such as EGFR and PDGFRA^28,29^. Hypermethylation following the IDH1^Mut^ may also lead to chromatin disorganization^30,31^ because mutated cells gain less condensed chromatin, leading to an increase in DNA damage^32^. On the other hand, IDH1^Mut^ decreases self-renewal and proliferation rate in vitro and in vivo in glioma cells^31,33^. This can be explained by hypermethylation and downregulation of PROM1 that encodes for CD133, the bona fide stem cell marker of glioma. CD133 is the primary stem cell factor in glioma, and a determiner of growth for glioblastoma cells in immunocompromised mice, a fact that was reported to be somewhat glioma specific in the context of IDH1 mutated cancers^12^. Phosphoinositide 3-kinase (PI3K)/AKT signaling is an important pathway for cell cycling and cell survival^34^. IDH1^Mut^ inhibits the PIK3/AKT signaling in human glioma cells^35^. This helps further explain the declining proliferation rate of glioma cells with IDH1^wt/R132H^ compared to IDH ^wt/wt^ cells^36^. Furthermore, by overexpression of IDH1^wt/R132H^ in the NSC derived from human-induced pluripotent stem cells (hiPSCs), Modrek et al. showed downregulation of expression of Sox2. Sox2 is largely responsible for NSC self-renewal and multipotency. Downregulation of Sox2 decreases the expression of Sox1, neuroD, and NGN2, which are important genes in differentiation to neural cells. Interestingly, Modrek et al.^31^ have revealed that overexpression of the P53 mutation can rescue these dysfunctions. In addition, activation of PI3K/AKT signaling is associated with aggressive human glioma^37^. PI3K/AKT signaling regulates downstream genes such as podoplanin (PDPN) and retinol-binding protein 1 (RBP1). PDPN plays a considerable role in glioma pathogenesis^34^. Downregulation of PDPN has been observed in glioma carrying IDH1 mutations, which can increase chances for survival in patients carrying IDH1^Mut^ ^38^. Moreover, an IDH1^Mut^ enhances AKT/mTOR activity. Since AKT/mTOR signaling is associated with cell migration, it may explain the high migration of IDH1^Mut^ glioma cells^36^.

### Clinical prognostic value of IDH1^Mut^

The IDH1 mutation, O6-methylguanine-DNA methyltransferase (MGMT) promoter methylation, and 1p 19q deletion have been introduced^39^ as markers for a low-grade glioma, anaplastic oligoastrocytoma, and glioblastoma, respectively, to predict sensitivity to chemotherapy and develop the appropriate prognosis. Patients who are positive for these three markers have a better progression-free survival^36^. After studying 1,010 patients, Hartmann et al.^40^ concluded that IDH1 mutations of the R132C type are strongly associated with astrocytoma, while IDH2 mutations mostly occur in oligodendroglial tumors. In addition, the IDH1 mutation occurs in younger patients^39^ which can be considered a predictor for grades 2 and 3, but not grade 4, glioma with a favorable prognosis, especially with radiation or alkylating therapy^39,40^. In this regard, the clinical trial demonstrated that patients with IDH1 mutations in grade 3 have a better response to chemotherapy, although chemo-radiotherapy may have a more positive effect on grades 2 and 3 tumors^41^.

### IDH1 Mut and immunology in neuro-oncology

**Fig. 1:**
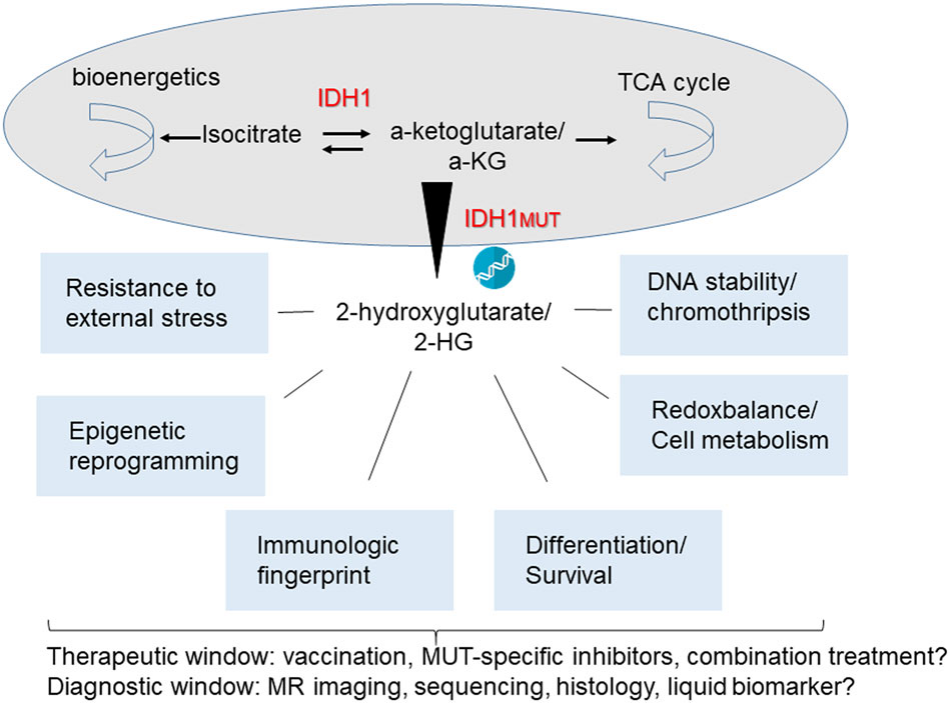
Schematic representation of the key enzymatic function of IDH1 and IDH1 mutant. IDH wild-type converts isocytrate into a-KG, whereas IDH1^Mut^ changes the enzymatic capacity to further convert a-KG into 2-HG, that accumulates inside the cell. Various cellular and molecular effects of D2HG accumulation have been identified amongst them the most prominent are depictured. Those changes open opportunities for IDH1^Mut^ specific therapeutic interventions and new diagnostic modalities.

IDH1-R132H is expressed in almost all slow-growing tumors, so the preparation of a vaccine targeting mutant IDH1 could be a novel therapeutic assay. Schumacher et al. found that IDH1-R132H contains an immunogenic epitope that can be used for vaccinations. They have constructed the artificial IDH1 polypeptides containing 15 amino acids. Injections of this construct with human major histocompatibility complexes (MHC) molecules in mice revealed that the mutated area could be presented on MHC class II that activated CD4^+^ T helper-1 and antibody production. This antibody can prevent the growth of IDH1^Mut^ tumor cells without inhibiting the normal functions of wt IDH1^42^. However, Weenink et al.^43^ reported that they failed to detect any antibody specific to IDH1-R132H or CD4^+^ T cell reactivity in sera of the LGG patients. In another study, Pellegatta et al. transplanted murine glioma cells containing the IDH1 mutation in the brains of mice and then immunized them to different peptides encompassing the IDH1 mutation. The immunized mice demonstrated an increasing number of CD8^+^ T cells and IFNγ production with an upregulation in the granzyme-b and perforin-1 and a corresponding downregulation of TGF-B2 and IL10. They also detected an antibody against IDH1^Mut^ ^44^. It has been shown that increasing the number of T cells may be due to a reduction of the expression of programmed death-ligand 1 (PD-L1) in mutant IDH^45^. However, Kohanbash et al.^46^ have found that reduction of PD-L1 may not result in a stronger T cell response, because D2HG inhibits ATP-dependent T cell receptor signaling, which in turn leads to suppression of T cell anti-tumor immunity. A summary of the main aspects of IDH1/IDH1^MUT^ in glioma cell biology are depictured in Fig. 1.

### Clinical trials targeting IDH1 in brain tumors

There are many experiments to discover new therapeutic medications such as small molecules or drugs to target IDH1/2 in low-grade gliomas conducted and some have progressed to validation in humans. These clinical trials are summarized in Table 1.

**Table 1:** Clinical trial of drugs or small molecules on gliomas containing the IDH1 mutation.

Clinical trail.gov	Phase of trial	Compound/treatment tool	Participants	Dosing	Result	Refs.
NCT02454634	I	IDH1 20 mer peptide vaccine (NOA-16)	39	8 times every 2/4 weeks	Cytotoxic immune response in and humoral immune responses	^79^
NCT02073994	I	AG-120	170	28-day cycles, oral	Ongoing trail	^80^
NCT02968940	II	Avelumab + Radiation	43	Every 2 weeks	–	^81^
NCT03212274	II	Olaparib	145	28-day cycles, oral	–	^81^
NCT02746081	I	BAY1436032	81	21-day cycles, oral	–	^82^
NCT02771301	I	IDH-R132H-DC vaccine	30	–	–	^83^
NCT02481154	I	AG-881 (ivosidenib)	95	28-day cycles, oral	Favorable safty at doses 100 mg and above	^84^
NCT03343197	II	AG-120 + AG-881	49	28-day cycles, oral	Ongoing trail	^85^
NCT02273739	I/III	AG-221	21	28-day cycles, oral	–	^86^
NCT02381886	I	IDH305	166	–	–	^87^
NCT02193347	I	IDH1 peptide vaccine	24	–	–	^88^
NCT03681028	I	AG-881		Oral	Tumor growth 6.8% compared to 24.5% placebo	^89^
NCT02209428	II	Temozolomide	54	Every 21 days, 6 cycles.	–	^90^
NCT03557359	II	Nivolumab	37	Every 2 weeks for 8 cycles for 2 years, infusion	Ongoing trail	^91^
NCT03960502	I	AG-881	5	Single dose, oral followed with infusion	–	^92^
NCT04164901	III	AG-881	366	Daily dosing, oral	Ongoing trail	^93^
NCT03684811	I/II	FT-2102 + Azacitidin	200	–	Ongoing trail	^94^
NCT03666559	II	Azacitidine	63	Seven consecutive days every 4 weeks, injection	–	^95^

## Modeling glioma carrying IDH1 mutations

There are many studies about the role of IDH1 and R132H mutation in glioma. However, a more complete understanding of the contribution of this mutation in tumorigenesis, the identification of interconnecting pathways, and the exploitation of IDH^Mut^-associated molecular and cellular alterations for diagnostic and therapeutic strategies have been hindered largely due to the lack of an appropriate model. One of the best ways to investigate different aspects of the role of the IDH1-R132H mutation is, providing in vitro and in vivo models. Since in vivo animal models are complex, expensive and time consuming, as well as ethically controversial, the establishment of in vitro models to study human tumorigenesis could be helpful.

In the view of the authors, one promising strategy is the generation of reductionistic molecular conditions that provide a tool to focus on specific aspects of tumor biology in an isogenic-controlled background. This reductionist model might be simple to study the effect of a single or multiple mutation(s) or mechanistic function and epigenetic modifications of these gene mutations in cancer. Other kinds of experimental models are those that aim to recapitulate the human disease conditions with all complexities. The cell of origin, as well as the type of transformation strategy, influences the feasibility of performing straightforward and functional assays^21^. The benefits and limitations of both approaches need to be considered and put in the context of the individual research question. We will include our perspective in this regard in the following model presentations.

## Procedures of in vitro modeling

### Patient-derived disease models

In vitro modeling is an important procedure to understand developmental and disease mechanisms, as well as the preclinical testing of drugs. Glioma models carrying the IDH1^Mut^, in vitro, can be based on the establishment of primary patient cells or generating IDH1 mutated cell lines via gene engineering or editing. In the following section, we will summarize the main findings made with classical serum-based cancer line, neurosphere cell lines, or primary cell models.

*Classical serum-cultured glioblastoma cell lines as a receiver matrix for transforming elements*: Human glioma cell lines derived from patients have been used as a tool to produce glioma models. The two most well-known glioma cell lines are U87^47^ and U251^48^. In spite of the long time culture, the genetic aberrations from the original tumors retained in these cell lines. Sequencing of the genome of U87 revealed an enormous number of indels, copy number variations, and translocations, which are most likely due to the condition of the serum culture^49^. Therefore, regular cell line verification is required. Moreover, passaging these cells in the medium containing serum differentiates U87 more to astrocyte^21^. However, due to easy gene manipulation and rapid culturing and maintenance of the original genetic aberrations, this glioma cell line has been used in several drugs in vitro screenings^50^. U87 and U251 have also been used to overexpress IDH1 wt and R132H, as well as to study the mechanism in glioma and the behavior of the cells. Zhu et al. transfected U87 to overexpress IDH1 wt, IDH1-R132H (pCMV-TAG2B) by lipofectamine 2000. Overexpressed IDH1-R132H cells were more sensitive to 5-FU, meaning that this medication could increase apoptosis in mutated IDH1 due to the decreasing expression of NADPH and CGH as antioxidants^36^. Cui et al. also overexpressed IDH1 Wt and R132H (ligated in pLenti6.3-MCS-IRES2-EGFP) by lentivirus in the U87 and U251 cell lines and showed the correlation between IDH1 mutations and B-catenin/Wnt pathways. Wnt/β-Catenin pathways are involved in the metastasis of different cancers through the initiation of epithelial to mesenchymal transition^51^, including glioblastoma. Overexpression of Wnt/β-Catenin in U87-IDH1-R132H and tumor samples carrying the R132H mutation restored a decline of proliferation and a high apoptosis ratio, reducing both invasion and migration in vitro and in vivo in the mutated cells. They concluded that mutant IDH1 can play an anti-tumor role in glioma. Li et al. used the pLPCX vector to overexpress IDH1-wt, IDH1-R132H, and IDH2-R172K by retrovirus in U87 and U373. IDH1-R132H and IDH2-R172K cells were more sensitive to radiation. This behavior was associated with a high rate of apoptosis and oxidative stress.

*Primary cell models from patients suffering from tumors of IDH*^*Mut*^: Primary, low-grade glioma (LGG)-derived cells survive in a neurobasal medium containing EGF and bFGF^52^. Piaskowski et al. tried to expand primary glioma cells carrying IDH1 mutations. They reported that glioma cells with IDH1 mutations cannot be cultured in vitro because of their death in standard cell culture condition^53^. Understanding the mechanism of this phenomenon can help the scientific community find new therapeutic targets.

The neurosphere assay is the gold standard for keeping stem cells in brain tissue^54^ and can help achieve better cultivation of mutant IDH glioma cells. Nevertheless, the behavior of the neurosphere depends on the genetics and grade of the tumor. Sphere formation can be the result of the anchorage-independent growth that happens due to oxidative stress^55^. Stoczynska-Fidelus et al.^53^ revealed that 3D cell culture increases the survival and proliferation rate of glioma cells. In contrast, Tiburcio et al.^55^ have demonstrated that neurosphere formation in heterogenous IDH1-R132H reduces the concentration of D2HG, compared with adherent culture. Recently, Jacob et al.^56^ established a patient-derived glioblastoma organoid (GBO) platform that can recapitulate parental tumor in vitro according to histological features, cellular diversity, gene expression, and mutational profiles. They dissected fresh tumor specimens into small pieces and established GBOs using neurosphere conditions in a shaking culture environment. They could generate organoids for 96.4% IDH1-wt and 66.7% with IDH1 mutations for over 48 weeks with a similar gene expression of parental tumor markers. Although they were successful in generating and testing glioma cells derived from patients, the efficiency for mutated IDH1 was low in comparison to LGG with IDH1 wt^56^. They concluded that more optimization is required to establish IDH1 mutated organoids. On the other hand, patient glioma cells can be reprogrammed to induce pluripotent stem cells (iPSC) by the overexpression of Yamanaka factors Oct4, Sox2, Cmyc and KLF4^57^, or Oct4, Nanog, L28, and Sox2^58^. Recently, Liu et al. overexpressed Yamanaka’s transcription factors in LGG cells BTO1 (carrying the R132C mutation), BTO3 (carrying the R132H mutation), and BT-142 (ATCC #ACS-1018) that originated from a grade III oligoastrocytoma carrying the mono-allelic R132H mutation. Their results demonstrate that cells containing the IDH1 mutation are resistant to reprogramming. Their explanation is the high sensitivity of the pluripotent stem cells to D2HG^59^ because when human embryonic stem cells (hESCs) were treated with D2HG for 24 h, the hESCs could not tolerate D2HG and die. They found, however, that reprogrammed colonies showed genetic changes, such as amplification of chromosome Xq23 or chromosome 11 and deletion of chromosome 1p, 4, 19q, and Y, amplification of 1.4 Mb region on chromosome 7q31, with co-amplification of genes located in this region. Analysis of tumor cells derived from LGG samples in a serum-free medium showed that they carry Xq23, 7q31 amplification, 1p 19q deletion, and the IDH1^Mut^, from which it can be concluded that these amplifications or deletions in primary patient cells carrying the IDH1^Mut^ are perhaps early mutational events associated with the manifestation of the IDH1^Mut^^59^.

Cell lines expressing mutated IDH1 can be generated by gene targeting methods such as zinc finger (ZFN), TALEN (Transcription activator-like effector nuclease), or clustered, regularly interspaced short palindromic repeats (CRISPR) or by overexpressing of wild-type and IDH1-R132H in various cell lines. The background of different experimental strategies, such as cellular background, mutation strategy, model validation, and core principal observations to model gliomas carrying the IDH1 mutation is summarized in Tables 2–4, respectively.

**Table 2:** Listing/summary of model generation strategies using overexpression of IDH^MUT^ on wild-type background.

Type of cells	Method	Vector	Protein or metabolite validation	Results of IDH1 mutation	References
*Overexpression: I. Transduction*					
IMA	Lentivirus	pLenti6.3/TO/V5-EST	MS	Suppress anchorage-independent growth	^55^
U87MG and U251	Lentivirus	pLenti6.3-MCS-IRES2-EGFP	WB	Reduces proliferation, cell survival, and invasion of human glioma by downregulating Wnt/-catenin signaling	^51^
U87MG and U373MG	Retrovirus	pLPCX vector	MS	Sensitivity to radiation therapy	^11^
hESC-NSC	Lentivirus	pEF1α-IDH1/pEF1α-R132H	MS	IDH1 combined with P53, and ATRX mutation block differentiation	^31^
hiPSC-NPC (ebiNSc)	Lentivirus	pLEX_307 plasmid gateway tec	WB, ICC	Sensitivity to radiation	^61^
IMA cell (IDH1 mutated astrocytoma)	Lentivirus	pLenti6.2/V5	MS	IDH1 mutation produce D2HG	^18^
GP2-293 cells	Retrovirus	pCMV-Sport6	MS	Blocking differentiation	^96^
Immortalized human astrocytes	Lentivirus	pLNCX2	MS	Hypermethylation	^62^
NHA-hTERT. HeLa, HT1080 and 293E	Retrovirus	pLPCX	MS	mTOR activation	^66^
U87, NHA	Lentivirus	–	WB	Increases RAD51-mediated HR and TMZ resistance	^97^
U87, HCT116, BT-142, T98G, LN229	Retrovirus	pLPCX	WB	Lower level of MCI-1 protein	^98^
U87 and U251	Lentivirus	PGC-FU-GFP	–	Decreasing GSH and increasing ROS	^99^
Mesenchymal stem cell	Lentivirus	pDONR221	MS	Dysregulate differentiation	^100^
SF10602, BT-142, GM847, MGG119	Lentivirus	GFP-tagged RAP1 or RFP-tagged XRCC1	WB	Telomeric dysfunction and alters DNA repair	^101^
U87	Lentivirus	U87, NHA	WB	Inhibition PIK3mTOR and reduction D2HG	^102^
*II. Transfection*					
U87	Transfection	p-EGFP-C1	WB	Enhancement of chemotherapy Sensitivity	^103^
U87	Lipofectamin 3000	pCMV-GV23	WB	Increased levels of repressive H3K9me3, enhancement of chemotherapy Sensitivity	^73^
SVGp12 and U251	Lipofectamin	pCMV-Sport6	WB	Induced proliferation of glioma cells via NFkB in a HIF1-α manner	^104^
293T, U87MG, LN-18	Lipofectamine 2000	pCMV-6	MS	Mutated IDH1 produce D2HG	^20^
U87, U138	Geneticin	p-EGFP-C2-bio	MS	Decreasing proliferation of IDH1-R132H	^33^
U87MG	Lipofectamine 2000	pCMV-Tag2B	WB	Upregulating AKT-mTOR	^36^
LN229	Fugene 6	–	Enzymatic assay	Neo-enzymatic activity for D2HG production	^23^
Murine GL261, SB28	Lipofectamine 2000	–	MS	Suppress STAT1 and CD8^+^ T cell accumulation	^46^
HT1080	Lipofectamine 2000	pCMV-Sport6	MS	Oncometabolite production	^105^
U251, Hela	Calcium phosphate	pcDNA6	WB	Acetate accumulation	^106^

**Table 3:** Listing/summary of model generation strategies using gene targeting.

HeLa cells	CRISPR	gWIZ.Luciferase	MS	Deficient in DNA repair	^107^
Human iPSC	CRISPR		MS	–	^70^
SVG-10B1	CRISPR		MS	–	^64^
HCT116 cell line	HR		MS	IDH1-R132H alters DNA-methylation	^108^

**Table 4:** Listing of model generation strategies applying successful reprogramming of patient cells.

Patient glioma cells	Lentivirus	Yamanaka factors	–	LGG cells containing IDH1 mutation are not reprogrammable	^59^

### Healthy donor-derived disease models

Although the origins of glioma are not well understood, gliomas are generally thought to arise from the neural stem cells or the populations of progenitor cells^2^. Neural stem cells (NSCs) present a stable genome when they propagate as neurospheres in serum-free medium^60^. Rosiak et al. used neural progenitor cells derived from human-induced pluripotent stem cells (hiPSC-NPCs) to express IDH1-R132H as a glioma model. They induced hiPSC-NPCs by a lentiviral construct containing the R132H mutation under the EF1 promoter. Overexpression of IDH1-R132H in hiPSC-NPCs decreased the differentiation ability of these cells to neuronal and inhibited differentiation to glial cells^61^. In addition, they demonstrated that overexpression of IDH1-R132H enhanced apoptosis in induced hiPSC-NPCs and during differentiation^61^. Apoptosis of the human glioma cells containing IDH1-R132H can be considered as a tumor suppressor by downregulating Wnt/β-catenin signaling^51^.

Turcan et al. demonstrated that induction of immortalized human astrocytes (by pLNCX2in packaged in lentivirus) to overexpress IDH1-wt and R132H can reshape methylome in the induced cells but in two different manners. Expression of IDH1-wt caused hypomethylation, while expression of IDH1-R132H hypermethylated CpG island in proneural, similar to what was observed in low-grade glioma (LGG) containing the IDH1-R132H mutation. They showed histone modification, such as hypermethylation of H3K9me2, H3K27me3 involved in hypermethylation of DNA, and declination of TET2-dependent 5-hydroxymethylcytosine (5hmC) levels. In this study, a mechanistic basis for the accumulation of DNA-methylation that leads to hypermethylation of CpG island was explained^62^. Tiburcio et al. also showed that overexpression of heterozygous IDH1-R132H by lentivirus (pLenti6.3/TO/V5-EST) in IMA can reduce the glutathione-oxidized glutathione (GSH/GSSG) ratio in comparison to IDH1 hemizygous and wt neurospheres. Interestingly, this ratio was even lower in adherent IDH1-R132H cells in comparison to neurospheres. In this study, Tiburcio et al.^55^ concluded that reducing power can control neurosphere genesis and the Wt allele contributes to gaining reduction power. This result not only emphasized the effect of heterozygosity of IDH1 in D2HG production but also confirmed that the IDH1 mutation in glioma can have an anti-oncogenic role^63^, which is an early phenomenon in glioma development.

New recombinant genomic techniques, TALEN and CRISPR, have been used to generate IDH1 mutant cell lines or study the behavior of IDH1 mut. Wei et al. employed a modified CRISPR/Cas9 to generate heterozygous IDH1-R132H mutation in human astroglial cells by applying single base editing. They revealed methylome changes associated with downregulation or upregulation of particular genes. In this regard, many metabolic enzymes were downregulated via hypermethylation of the promoter, including acyl-CoA dehydrogenase (ACADS), Aldehyde Dehydrogenase 2 Family (ALDH2), and Aldehyde Oxidase 1 (AOX1), indicating the involvement of IDH1 in multiple energy pathways. In addition, they showed that hypermethylation of the promoter can lead to histone modification and declination of histone markers such as H3K27me3, H3K36me3, and H3K4me3 in IDH1-R132H cells^64^. Wei et al. also demonstrated increasing migration of cells by upregulation of integrin β4 (ITGB4) and inhibition of the proliferation of mutant cells. Furthermore, Wei et al. introduced Yes-associated protein (YAP) and Notch signaling, two prominent phylogenetically conserved stem cell pathways, as a molecular target responsible for cell growth inhibition in IDH1-R132H cells. In this study, Wei et al.^64^ also showed that enhancement of the migration and invasion of IDH1-R132H cells promote dependence on D2HG.

Nowadays, hiPSC is used as a promising tool in disease modeling^65^. Human iPSC can generate the proper model for glioma modeling because of self-renewing and differentiation to different types of cells^66^. Nevertheless, the genome instability and epigenetic memory during the reprogramming process and iPSC maintenance remain unexplained. Due to the toxicity of D2HG on hiPSCs^59^, scientists should investigate ways to by-pass this issue.

2D disease modeling by human iPSC can show some disease phenotypes, but there are no tissue or organ level structures. It is supposed that 3D modeling can demonstrate accurate pathophysiology of disease^67^ and can recapitulate the in vivo environment^52^ because, in this condition, cells can express stemness genes. Laks et al. have shown that the spheroid culture of glioma primary cells expresses new genes related to malignancy and may help to culture primary cells carrying the IDH1 mutation. It is possible, however, that all spheres from primary glioma cells do not show a correlation with the parental tumor^68^. Cerebral organoids derived from hiPSCs are a promising way to recapitulate diseases in vitro, such as GBM. Bian et al.^69^ established organoids expressing MYC-amplification (MYCOE), and organoids carrying CDKN2A-/CDKN2B-/EGFROE/EGFRvIIIOE, NF1-/PTEN-/TP53- (p53), and EGFRvIIIOE/ CDKN2A-/PTEN- called neoplastic cerebral organoids (neoCOR). Engraftment of neoCORs upon the renal subcapsular showed retaining and expansion of the organoid in the renal capsule with poor differentiation, as well as recapitulate tumorigenesis in vivo. NeoCORs could interact with normal organoids and tissue in the renal capsule, which would verify the invasiveness of the gene-engineered organoids^69^.

Recently, Köpp et al.^70^ reported the gene modification of IDH1-R132H in hiPSC by CRISPR/Cas9 (efficiency 1%). In light of the challenges associated with generating a CRISP-mediated, iPSC-based IDH1 model, it will be interesting to see what functional validations of this model will be provided in the future. Initial results show that cerebral organoids from gene-modified iPSC-R132H are not affected in cell differentiation in the maturation of cerebral organoids. There are, however, some differences in cell growth between organoid DH1-Wt and IDH1-R132H.

In addition, recent work proved that iPSC-based creation of synthetic models for glioblastoma is a relevant strategy to study pathophysiological relevant tumor progression and to create molecular subtype-specific identities. We argue that using such an experimental design is a promising strategy that should be exploited in the search for IDH1-specific chemotherapies.

### In vivo models

Most of our knowledge about the effect of IDH mutations in glioma were obtained from clinical studies or overexpression of the IDH1/2 mutation in various types of cells. Sasaki et al. generated the brain knock-in IDH1-R132H mouse model by the Cre-Laxp system. They induced mutated IDH1 in nestin-expressing cells and showed that this led to the death of mice directly after birth. Brain hemorrhage associated with accumulation of D2HG, high levels of hypoxia-inducible transcription factor-1a (HIF1α), reduction of ROS level, impaired collagen maturation, and disruption of basement formation were observed in mutant mice. They attributed these defects to the production of D2HG^71^. In another study, Bardella et al. generated a mouse model of glioma by condition expressing IDH1-R132H in the subventricular zone (SVZ) in the brains of adult mice. These mice showed hydrocephalus and expanded lateral ventricles with an accumulation of D2HG and a reduction of α-KG. NSCs isolated from these mice demonstrated a higher proliferation rate in vitro, and the SVZ area of the brain showed a high proliferation rate of these cells in vivo^72^. Recently, Nunez et al. generated a mouse model expressing IDH1-R132H associated with the deletion of p53 and ATRX. They showed an increase in the survival time without any treatment, as has been observed in patients. In addition, this study demonstrated hypermethylation of histone 3, which can cause epigenetic reprogramming and upregulation of the ataxia-telangiectasia-mutated (ATM) signaling pathway that leads to DNA damage response (DDR) as observed in the human glioma cells from surgical biopsies^32^. Moreover, the IDH1 mutation in this model resulted in radiotherapy resistance that could be restored by pharmacological inhibition of ATM or checkpoint kinases 1 and 2, essential kinases in the DDR. Translation of these findings to patients with IDH1-R132H glioma and p53 and ATRX loss could help the therapeutic efficacy of radiotherapy and patient survival^73^.

## Discussion: perspectives of the authors

To generate an in vitro model, the selection of a culture environment and the medium is a pivotal factor to recapitulate glioma. Since IDH1 mutations relate to both astrocytoma and oligodendroglioma, selecting the correct medium is essential to keep the IDH1 mutated model similar to the patient’s condition, because using an inaccurate medium can change the sensitivity of cells to different drugs. For example, using NSC media for oligodendroglioma patient cells caused an omission of oligo precursor cells (OPC) properties and obtained high sensitivity to temozolomide wheeze, OPC medium supplemented with platelet-derived growth factor (PDGF) sustained OPC proliferation and development in vitro.

Glioblastoma, like other cancers, is a polygenic disease. Understanding cell behavior, or glioma mechanism, or analyzing each gene’s role in glioma can help find the mechanism of glioma and targeting the pathway for treatment. On the other hand, one can combine the effect of multiple mutations related to glioma and understand the behavior of cells after these combinations. These models can be used as disease models to understand or screen various drugs on them. Human iPSCs are self-renewal cells with the potential to differentiate into multiple kinds of cells, and relative genome stability may be a promising tool to recapitulate the disease in vitro. Generating in vitro models by overexpression of the wild-type or mutated IDH1 can be a way to establish the glioma model containing IDH1-R132H, although this is not an accurate model for disease modeling. Overexpression of the gene by transduction or transfection is based on random integration, in which the copy numbers and the location of the integrated gene cannot be controlled. The latter may attenuate or interfere with the expression of integrated genes or activate the oncogenes.

Reprogramming of glioma cells derived from patients and established isogenic clones is an alternative way to generate disease models for glioma—with or without IDH1 mutations. In this way, several isogenic clones should be selected to confirm cell characterization and metabolite production. Since D2HG production from mutated IDH1/2 can be a factor in inhibiting the reprogramming process, designing small molecules that can attach to the active catalytic site of mutated IDH1/2 may inhibit the mutated enzyme from producing D2HG instead of α-KG. In this regard, IDH1 inhibitors (AG-120 ML 309^74^, AG-120^75^, and AGI-5198^76^), and IDH2 inhibitors (AG-221^77^, AGI-6780^78^) have shown that they can restore the IDH1 mutation effects^17^. Reprogrammed or overexpressed iPSCs or their derivations can differentiate and can be cultured in 2D or 3D. 3D cultures can recapitulate the organ structures. Brain organoids derived from human iPSCs (mini brain) are three-dimensional (3D) self-organized neural structures that can recapitulate the structure and development of the brain. Since cerebral organoids contain neuronal cells, astrocytes, and oligodendrocytes, they can be used to mimic astroglioma or oligodendroglioma. The high tendency of the cells carrying mutated IDH1 to grow as a sphere makes organoids a suitable model for understanding the pathophysiology of this mutation in glioma and may help us develop patient-specific therapies.

The fusion of cerebral organoids derived from iPSC and spheroid of patient glioma cells is a novel strategy to generate a glioblastoma model. Brain organoids may be able to fuse with glioma neurospheres or single glioma cells carrying the IDH1 mutation. This model can reveal the heterogenicity in glioma as well. Moreover, the interrogation of single-cell mutagenesis technologies in pluripotent or cancer cells targeting different disease-relevant genes, either in a multiplex approach in one cell or each selected gene in one cell, and subsequent co-culturing the differentially modulated cells in one organoid structure, while technologically realistic, remains a strategy on which little has been published to model the intra-tumoral heterogeneity of cancer in vitro.

One of the issues of organoid modeling is a short access to the vascular system. The lack of vascularization makes it very difficult for inner cells to have access to nutrients and gas exchange, which causes necrotic cells in the center of the organoid. Co-cultures of fused organoids or gene-modified organoids with differentiated endothelial cells may solve this problem in disease modeling. Furthermore, using hydrogels can increase the diffusion of the nutrients and oxygen through organoid or 3D structures.

Co-cultures of patients, healthy T cells, or microglial or innate immune cells with fused organoids or organoid carrying mutated IDH1 can recapitulate cell infiltration in the brain. Another way to help generate the IDH1-R132H model could be through the transplantation of mutated IDH1 organoids in the brain of host animals as “in vivo bioreactors” to support the growth of cerebral organoids. Transplanted human brain organoids can differentiate neural and neuronal cells and integrate after transplantation in the cortex of mice. Infiltration of microglial, axon prolongation, and vascularization of the grafted organoids can also recapitulate the glioma. With growing evidence of the usefulness, and perhaps even the superiority of these modern cell culture technologies compared to animal models, we anticipate that the field will adopt those approaches as standard operating procedures in the near future. This is well in line with ethical considerations in science currently driving the 3R movement.

## References

[1] Ludwig, K. & Kornblum, H. I. Molecular markers in glioma. *J. Neuro-Oncol.*10.1007/s11060-017-2379-y (2017). 10.1007/s11060-017-2379-y PMC5568999 28233083

[2] Brat DJ (2015). Comprehensive, integrative genomic analysis of diffuse lower-grade gliomas. N. Engl. J. Med..

[3] Foote MB, Papadopoulos N, Diaz LA (2015). Genetic classification of gliomas: refining histopathology. Cancer Cell.

[4] Huang J (2019). Isocitrate dehydrogenase mutations in glioma: From basic discovery to therapeutics development. Front. Oncol..

[5] Kloosterhof NK, Bralten LBC, Dubbink HJ, French PJ, van den Bent MJ (2011). Isocitrate dehydrogenase-1 mutations: a fundamentally new understanding of diffuse glioma?. Lancet Oncol..

[6] Krell D (2011). Screen for IDH1, IDH2, IDH3, D2HGDH and l2HGDH mutations in glioblastoma. PLoS ONE.

[7] Sjöblom T (2006). The consensus coding sequences of human breast and colorectal cancers. Science.

[8] Parsons, D. W. et al. An integrated genomic analysis of human glioblastoma multiforme. *Science* **321**, 1807 (2008). 10.1126/science.1164382 PMC2820389 18772396

[9] Singh A, Gurav M, Dhanavade S, Shetty O, Epari S (2017). Diffuse glioma—rare homozygous IDH point mutation, is it an oncogenetic mechanism?. Neuropathology.

[10] Mu, L. et al. IDH1 R132H mutation is accompanied with malignant progression of paired primary-recurrent astrocytic tumours. *J. Cancer* **8**, 2704–2712 (2017). 10.7150/jca.20665 PMC5604202 28928859

[11] Li S (2013). Overexpression of isocitrate dehydrogenase mutant proteins renders glioma cells more sensitive to radiation. Neuro. Oncol..

[12] Unruh D (2019). Methylation and transcription patterns are distinct in IDH mutant gliomas compared to other IDH mutant cancers. Sci. Rep..

[13] Horbinski C (2013). What do we know about IDH1/2 mutations so far, and how do we use it?. Acta Neuropathol..

[14] Amankulor NM (2017). Mutant idh1 regulates the tumor-associated immune system in gliomas. Genes Dev..

[15] Al-Khallaf H (2017). Isocitrate dehydrogenases in physiology and cancer: biochemical and molecular insight. Cell Biosci..

[16] Martínez-Reyes I, Chandel NS (2020). Mitochondrial TCA cycle metabolites control physiology and disease. Nat. Commun..

[17] Kaminska B, Czapski B, Guzik R, Król SK, Gielniewski B (2019). Consequences of IDH1/2 mutations in gliomas and an assessment of inhibitors targeting mutated IDH proteins. Molecules.

[18] Jin G (2013). Disruption of wild-type IDH1 suppresses D-2-hydroxyglutarate production in IDH1-mutated gliomas. Cancer Res..

[19] Garrett M (2018). Metabolic characterization of isocitrate dehydrogenase (IDH) mutant and IDH wildtype gliomaspheres uncovers cell type-specific vulnerabilities. Cancer Metab..

[20] Dang L (2009). Cancer-associated IDH1 mutations produce 2-hydroxyglutarate. Nature.

[21] Robertson, F. L., Marqués-Torrejón, M. A., Morrison, G. M. & Pollard, S. M. Experimental models and tools to tackle glioblastoma. *DMM Dis. Model. Mech*. **12**, dmm040386 (2019). 10.1242/dmm.040386 PMC6765190 31519690

[22] Yan H (2009). Mutations in gliomas. N. Engl. J. Med..

[23] Pusch S (2014). D-2-hydroxyglutarate producing neo-enzymatic activity inversely correlates with frequency of the type of isocitrate dehydrogenase 1 mutations found in glioma. Acta Neuropathol. Commun..

[24] Linninger A (2018). Modeling the diffusion of D-2-hydroxyglutarate from IDH1 mutant gliomas in the central nervous system. Neuro. Oncol..

[25] Ward PS (2013). The potential for isocitrate dehydrogenase mutations to produce 2-hydroxyglutarate depends on allele specificity and subcellular compartmentalization. J. Biol. Chem..

[26] Kranendijk M, Struys EA, Salomons GS, Van der Knaap MS, Jakobs C (2012). Progress in understanding 2-hydroxyglutaric acidurias. J Inherit Metab Dis.

[27] Yen KE, Bittinger MA, Su SM, Fantin VR (2010). Cancer-associated IDH mutations: biomarker and therapeutic opportunities. Oncogene.

[28] Sun Y (2014). A glioma classification scheme based on coexpression modules of EGFR and PDGFRA. Proc. Natl Acad. Sci. USA.

[29] Raineri S, Mellor J (2018). IDH1: linking metabolism and epigenetics. Front. Genet..

[30] Flavahan WA (2016). Insulator dysfunction and oncogene activation in IDH mutant gliomas. Nature.

[31] Modrek AS (2017). Low-grade astrocytoma mutations in IDH1, P53, and ATRX cooperate to block differentiation of human neural stem cells via repression of SOX2. Cell Rep..

[32] Núñez FJ (2019). IDH1-R132H acts as a tumor suppressor in glioma via epigenetic up-regulation of the DNA damage response. Sci. Transl. Med..

[33] Bralten LBC (2011). IDH1 R132H decreases proliferation of glioma cell lines in vitro and in vivo. Ann. Neurol..

[34] King D, Yeomanson D, Bryant HE (2015). PI3King the lock: targeting the PI3K/Akt/mTOR pathway as a novel therapeutic strategy in neuroblastoma. J. Pediatr. Hematol./Oncol..

[35] Tateishi K (2019). PI3K/AKT/mTOR pathway alterations promote malignant progression and xenograft formation in oligodendroglial tumors. Clin. Cancer Res..

[36] Zhu H (2017). IDH1 R132H mutation enhances cell migration by activating AKT-mTOR signaling pathway, but sensitizes cells to 5-FU treatment as NADPH and GSH are reduced. PLoS ONE.

[37] Peterziel H (2012). Expression of podoplanin in human astrocytic brain tumors is controlled by the PI3K-AKT-AP-1 signaling pathway and promoter methylation. Neuro. Oncol..

[38] Sun, C. et al. Wild-type IDH1 and mutant IDH1 opposingly regulate podoplanin expression in glioma. *Transl. Oncol*. **13**, 100758 (2020). 10.1016/j.tranon.2020.100758 PMC7097522 32208352

[39] Chen X (2019). Clinical prognostic value of isocitrate dehydrogenase mutation, O-6-methylguanine-DNA methyltransferase promoter methylation, and 1p19q co-deletion in glioma patients. Ann. Transl. Med..

[40] Hartmann C (2009). Type and frequency of IDH1 and IDH2 mutations are related to astrocytic and oligodendroglial differentiation and age: a study of 1,010 diffuse gliomas. Acta Neuropathol..

[41] Dang L, Yen K, Attar EC (2016). IDH mutations in cancer and progress toward development of targeted therapeutics. Ann. Oncol..

[42] Schumacher T (2014). A vaccine targeting mutant IDH1 induces antitumour immunity. Nature.

[43] Weenink B (2019). Lack of B and T cell reactivity towards IDH1R132H in blood and tumor tissue from LGG patients. J. Neurooncol..

[44] Pellegatta S (2015). Effective immuno-targeting of the IDH1 mutation R132H in a murine model of intracranial glioma. Acta Neuropathol. Commun..

[45] Han S (2020). IDH mutation in glioma: molecular mechanisms and potential therapeutic targets. Br. J. Cancer.

[46] Kohanbash G (2017). Isocitrate dehydrogenase mutations suppress STAT1 and CD8+ T cell accumulation in gliomas. J. Clin. Invest..

[47] Pontén J, Macintyre EH (1968). Long term culture of normal and neoplastic human glia. Acta Pathol. Microbiol. Scand..

[48] Westermark B, Pontén J, Hugosson R (2009). Determinants for the establishment of permanent tissue culture lines from human gliomas. Acta Pathol. Microbiol. Scand. Sect. A Pathol..

[49] Lenting K, Verhaak R, ter Laan M, Wesseling P, Leenders W (2017). Glioma: experimental models and reality. Acta Neuropathol..

[50] Mikhailova V (2018). Towards an advanced cell-based in vitro glioma model system. AIMS Genet..

[51] Cui D (2016). R132H mutation in IDH1 gene reduces proliferation, cell survival and invasion of human glioma by downregulating Wnt/β-catenin signaling. Int. J. Biochem. Cell Biol..

[52] Ledur PF, Onzi GR, Zong H, Lenz G (2017). Culture conditions defining glioblastoma cells behavior: what is the impact for novel discoveries?. Oncotarget.

[53] Piaskowski S (2011). Glioma cells showing IDH1 mutation cannot be propagated in standard cell culture conditions. Br. J. Cancer.

[54] Singec I (2006). Defining the actual sensitivity and specificity of the neurosphere assay in stem cell biology. Nat. Methods.

[55] Tiburcio PDB (2018). Functional requirement of a wild-type allele for mutant IDH1 to suppress anchorage-independent growth through redox homeostasis. Acta Neuropathol..

[56] Jacob F (2020). A patient-derived glioblastoma organoid model and biobank recapitulates inter- and intra-tumoral heterogeneity. Cell.

[57] Takahashi K (2007). Induction of pluripotent stem cells from adult human fibroblasts by defined factors. Cell.

[58] Yu, J. et al. Induced pluripotent stem cell lines derived from human somatic cells. *Science* **318**, 1917–1920 (2007). 10.1126/science.1151526 18029452

[59] Liu Z (2019). Characterization of iPSCs derived from low grade gliomas revealed early regional chromosomal amplifications during gliomagenesis. J. Neurooncol..

[60] Reynolds BA, Weiss S (1992). Generation of neurons and astrocytes from isolated cells of the adult mammalian central nervous system. Science.

[61] Rosiak K (2016). IDH1R132H in neural stem cells: differentiation impaired by increased apoptosis. PLoS ONE.

[62] Turcan S (2012). IDH1 mutation is sufficient to establish the glioma hypermethylator phenotype. Nature.

[63] Huang LE (2017). IGFBP2 expression predicts IDH-mutant glioma patient survival. Oncotarget.

[64] Wei S (2018). Heterozygous IDH1 R132H/WT created by “single base editing” inhibits human astroglial cell growth by downregulating YAP. Oncogene.

[65] Bassett AR (2017). Editing the genome of hiPSC with CRISPR/Cas9: disease models. Mamm. Genome.

[66] Carbonneau, M. et al. The oncometabolite 2-hydroxyglutarate activates the mTOR signalling pathway. *Nat. Commun*. **7**, 12700 (2016). 10.1038/ncomms12700 PMC5027283 27624942

[67] Liu C, Oikonomopoulos A, Sayed N, Wu JC (2018). Modeling human diseases with induced pluripotent stem cells: From 2D to 3D and beyond. Dev.

[68] Laks DR (2016). Large-scale assessment of the gliomasphere model system. Neuro. Oncol..

[69] Bian, S., Repic, M., Guo, Z., Kavirayani, A. & Burkard, T. Europe PMC Funders Group Genetically engineered cerebral organoids model brain tumour formation. *Nat Methods* **15**, 631–639 (2019). 10.1038/s41592-018-0070-7 PMC6071863 30038414

[70] Köpp, A et al. OS12.1 Editing of IDH1 R132H mutation in human induced pluripotent stem cells to investigate tumor genesis in glioma. *Neuro-Oncology* **21**, iii22. https://academic.oup.com/neuro-oncology/article-abstract/21/Supplement_3/iii22/5564391?redirectedFrom=fulltext (2019).

[71] Sasaki M (2012). D-2-hydroxyglutarate produced by mutant Idh1 perturbs collagen maturation and basement membrane function. Genes Dev..

[72] Bardella C (2016). Expression of Idh1R132H in the murine subventricular zone stem cell niche recapitulates features of early gliomagenesis. Cancer Cell.

[73] Yin N (2020). IDH1-R132H mutation radiosensitizes U87MG glioma cells via epigenetic downregulation of TIGAR. Oncol. Lett..

[74] Davis, M. *Probe Report: ML309, A Potent Inhibitor of R132H Mutant IDH1 Capable Of Reducing 2-Hydroxyglutarate Production in U87 MG Glioblastoma Cells* 1–29 (National Center for Biotechnology Information, Bethesda, 2010). 23905201

[75] Burris, H. et al. Abstract PL04-05: The first reported results of AG-120, a first-in-class, potent inhibitor of the IDH1 mutant protein, in a Phase I study of patients with advanced IDH1-mutant solid tumors, including gliomas. *Mol. Cancer Therap.* 14, PL04-05-PL04-05 (2015).

[76] Rohle D (2013). An inhibitor of mutant IDH1 delays growth and promotes differentiation of glioma cells. Science.

[77] Fan, B. Pharmacokinetic/pharmacodynamic evaluation of AG-120, a potent inhibitor of the IDH1 mutant protein, in a phase 1 study of IDH1-mutant advanced hematologic malignancies. EHA Library. 100713. https://library.ehaweb.org/eha/2015/20th/100713/bin.fan.pharmacokinetic.pharmacodynamic.evaluation.of.ag-120.a.potent.html?f=m1 (2015).

[78] Wang F (2013). Targeted inhibition of mutant IDH2 in leukemia cells induces cellular differentiation. Science.

[79] NIH. Search of: NCT02454634 - List Results - ClinicalTrials.gov. https://clinicaltrials.gov/ct2/results?cond=&term=NCT02454634&cntry=&state=&city=&dist=.

[80] NIH. Search of: NCT02073994 - List Results - ClinicalTrials.gov. https://clinicaltrials.gov/ct2/results?cond=&term=NCT02073994&cntry=&state=&city=&dist=.

[81] NIH. Search of: NCT03212274 - List Results - ClinicalTrials.gov. https://clinicaltrials.gov/ct2/results?cond=&term=NCT03212274&cntry=&state=&city=&dist=.

[82] NIH. Search of: NCT02746081 - List Results - ClinicalTrials.gov. https://clinicaltrials.gov/ct2/results?cond=&term=NCT02746081&cntry=&state=&city=&dist=.

[83] NIH. Search of: NCT02771301 - List Results - ClinicalTrials.gov. https://clinicaltrials.gov/ct2/results?cond=&term=NCT02771301&cntry=&state=&city=&dist=.

[84] NIH. Search of: NCT02481154 - List Results - ClinicalTrials.gov. https://clinicaltrials.gov/ct2/results?cond=&term=NCT02481154&cntry=&state=&city=&dist=.

[85] NIH. Search of: NCT03343197 - List Results - ClinicalTrials.gov. https://clinicaltrials.gov/ct2/results?cond=&term=NCT03343197&cntry=&state=&city=&dist=.

[86] NIH. Search of: NCT02273739 - List Results - ClinicalTrials.gov. https://clinicaltrials.gov/ct2/results?cond=&term=NCT02273739&cntry=&state=&city=&dist=.

[87] NIH. Search of: NCT02381886 - List Results - ClinicalTrials.gov. https://clinicaltrials.gov/ct2/results?cond=&term=NCT02381886&cntry=&state=&city=&dist=.

[88] NIH. Search of: NCT02193347 - List Results - ClinicalTrials.gov. https://clinicaltrials.gov/ct2/results?cond=&term=NCT02193347&cntry=&state=&city=&dist=.

[89] NIH. Search of: NCT03681028 - List Results - ClinicalTrials.gov. https://clinicaltrials.gov/ct2/results?cond=&term=NCT03681028&cntry=&state=&city=&dist=.

[90] NIH. Search of: NCT02209428 - List Results - ClinicalTrials.gov. https://clinicaltrials.gov/ct2/results?cond=&term=NCT02209428&cntry=&state=&city=&dist=.

[91] NIH. Search of: NCT03557359 - List Results - ClinicalTrials.gov. https://clinicaltrials.gov/ct2/results?cond=&term=NCT03557359&cntry=&state=&city=&dist=.

[92] NIH. Search of: NCT03960502 - List Results - ClinicalTrials.gov. https://clinicaltrials.gov/ct2/results?cond=&term=NCT03960502&cntry=&state=&city=&dist=.

[93] NIH. Search of: NCT04164901 - List Results - ClinicalTrials.gov. https://clinicaltrials.gov/ct2/results?cond=&term=NCT04164901&cntry=&state=&city=&dist=.

[94] NIH. Search of: NCT03684811 - List Results - ClinicalTrials.gov. https://clinicaltrials.gov/ct2/results?cond=&term=NCT03684811&cntry=&state=&city=&dist=.

[95] NIH. Search of: NCT03666559 - List Results - ClinicalTrials.gov. https://clinicaltrials.gov/ct2/results?cond=&term=NCT03666559&cntry=&state=&city=&dist=.

[96] Lu C (2012). IDH mutation impairs histone demethylation and results in a block to cell differentiation. Nature.

[97] Ohba S, Mukherjee J, See WL, Pieper RO (2014). Mutant IDH1-driven cellular transformation increases RAD51-mediated homologous recombination and temozolomide resistance. Cancer Res..

[98] Karpel-Massler G (2017). Induction of synthetic lethality in IDH1-mutated gliomas through inhibition of Bcl-xL. Nat. Commun..

[99] Shi J (2015). Decreasing GSH and increasing ROS in chemosensitivity gliomas with IDH1 mutation. Tumor Biol..

[100] Jin Y (2015). Mutant idh1 dysregulates the differentiation of mesenchymal stem cells in association with gene-specific histone modifications to cartilage- and bone-related genes. PLoS ONE.

[101] Mukherjee J (2018). Mutant IDH1 cooperates with ATRX loss to drive the alternative lengthening of telomere phenotype in glioma. Cancer Res..

[102] Batsios G (2019). PI3K/mTOR inhibition of IDH1 mutant glioma leads to reduced 2HG production that is associated with increased survival. Sci. Rep..

[103] Wang JB, Dong DF, Wang MDE, Gao K (2014). IDH1 overexpression induced chemotherapy resistance and IDH1 mutation enhanced chemotherapy sensitivity in glioma cells in vitro and in vivo. Asian Pac. J. Cancer Prev..

[104] Wang G (2014). Mutation of isocitrate dehydrogenase 1 induces glioma cell proliferation via nuclear factor-κB activation in a hypoxia-inducible factor 1-α dependent manner. Mol. Med. Rep..

[105] Dexter JP (2018). Lack of evidence for substrate channeling or flux between wildtype and mutant isocitrate dehydrogenase to produce the oncometabolite 2-hydroxyglutarate. J. Biol. Chem..

[106] Koyasu S (2019). Increased 14C-acetate accumulation in IDH-mutated human glioblastoma: implications for detecting IDH-mutated glioblastoma with 11C-acetate PET imaging. J. Neurooncol..

[107] Sulkowski, P. L. et al. 2-Hydroxyglutarate produced by neomorphic IDH mutations suppresses homologous recombination and induces PARP inhibitor sensitivity. *Sci. Transl. Med*. **9**, eaal2463 (2017). 10.1126/scitranslmed.aal2463 PMC5435119 28148839

[108] Duncan CG (2012). A heterozygous IDH1R132H/WT mutation induces genome-wide alterations in DNA methylation. Genome Res..

